# Ferric Oxide Colloid: Towards Green Nano-Fertilizer for Tomato Plant with Enhanced Vegetative Growth and Immune Response Against Fusarium Wilt Disease

**DOI:** 10.1007/s10904-022-02442-6

**Published:** 2022-07-22

**Authors:** Sherif Elbasuney, Gharieb S. El-Sayyad, Mohamed S. Attia, Amer M. Abdelaziz

**Affiliations:** 1 Head of Nanotechnology Research Center, Military Technical College (MTC), Cairo, Egypt; 2 School of Chemical Engineering, Military Technical College (MTC), Cairo, Egypt; 3 Drug Microbiology Lab, Drug Radiation Research Department, National Centre for Radiation Research and Technology (NCRRT), Egyptian Atomic Energy Authority (EAEA), Cairo, Egypt; 4 Botany and Microbiology Department, Faculty of Science, Al-Azhar University, Cairo, Egypt

**Keywords:** Tomato plant, *Fusarium oxysporum*, Ferric oxide colloid, Systemic resistance, Antifungal activity

## Abstract

Global food crisis due to climate change, pandemic COVID-19 outbreak, and Russia-Ukraine conflict leads to catastrophic consequences; almost 10 percent of the world’s population go to bed hungry daily. Narrative solution for green agriculture with high vegetation and crop yield is mandatory; novel nanomaterials can improve plant immunity and restrain plant diseases. Iron is fundamental nutrient element; it plays vital role in enzyme activity and RNA synthesis; furthermore it is involved in photosynthesis electron-transfer chains. This study reports on the facile synthesis of colloidal ferric oxide nanoparticles as novel nano-fertilizer to promote vegetation and to suppress *Fusarium* wilt disease in tomato plant. Disease index, protection percent, photosynthetic pigments, and metabolic indicators of resistance in plant as response to induction of systemic resistance (SR) were recorded. Results illustrated that Fe_2_O_3_ NPs had antifungal activity against *F*. *oxysporum*. Fe_2_O_3_ NPs (at 20 µg/mL) was the best treatment and reduced percent disease indexes by 15.62 and gave highly protection against disease by 82.15% relative to untreated infected plants. Fe_2_O_3_ NPs treatments in either (non-infected or infected) plants showed improvements in photosynthetic pigments, osmolytes, and antioxidant enzymes activity. The beneficial effects of the synthesized Fe_2_O_3_ NPs were extended to increase not only photosynthetic pigments, osmolytes contents but also the activities of peroxidase (POD), polyphenol oxidase (PPO), catalase (CAT) and superoxide dismutase (SOD), enzymes of the healthy and infected tomato plants in comparison with control. For, peroxidase and polyphenol oxidase activities it was found that, application of Fe_2_O_3_ NPs (10 µg/mL) on challenged plants offered the best treatments which increased the activities of POD by (34.4%) and PPO by (31.24%). On the other hand, application of Fe_2_O_3_ NPs (20 µg/mL) on challenged plants offered the best treatments which increased the activities of CAT by (30.9%), and SOD by (31.33%).

**Supplementary Information:**

The online version contains supplementary material available at 10.1007/s10904-022-02442-6.

## Introduction

COVID-19 pandemic, climate change, and Russia-Ukraine conflict contributed to the surge of food cost. According to world food program, there are 44 million people in 38 countries at the ‘emergency’ phase of food insecurity [1, 2]. Novel approaches for green agriculture with enhanced plant immunity, vegetative growth, and crop productivity are highly appreciated [3]. Wilt disease, caused by the soil pathogen *Fusarium oxysporum,* has a negative impact on plant growth, metabolic properties, and crop yield. Consequently crop quality and quantity could be decreased significantly [4–8]. It should be considered that; some fungal species can create a harmful impact on crops and plants. Fusarium wilt disease is a systemic disease, as the fungus spreads inside the infected plant. It is difficult to combat Fusarium wilt disease chemically [9]. This disease is very dangerous, especially in areas where the hot weather prevails during the planting season [10]. In addition, plant malnutrition is one of the most serious problems that threaten agricultural wealth; as it causes huge losses in agricultural production, reduction in the product quality, as well as the secretion of toxins that cause poisoning and multiple serious diseases affecting humans and animals that feed on this product [11]. The plant may be exposed to a series of oxidative explosions in the cells, and the enzymes do not perform important chemical transformations to protect it from oxidative explosions, causing cell death and the susceptibility to infection with pathogens may increase [12].

Tomato is one of the most important vegetable crop in Egypt and it’s grown all year round in Egypt. However, production faces some problems in summer season due to high temperature and insect born viruses diseases prevailing in this time period [13].

Fertilizers are defined as natural or synthetic materials that provide the plant with nutrients necessary for its growth, development, and crop production. Depending on their source, fertilizers are classified into two main categories, including organic (natural) and chemical (synthetic) fertilizers [14]. Researchers found that plants treated with nano fertilizers and natural bio-stimulants, tends to have more activities of antioxidant enzymes [15]. Nanotechnology could play an important role in agriculture. The potential uses and benefits of nanotechnology are enormous and can be exploited to improve production and resistance to plant diseases [16]. Nanotechnology enables plants to exploit water, pesticides, and fertilizers more efficiently [17]. Nanotechnology (polymer/inorganic nanocomposites) may bring potential benefits via improving plant immunity, disease resistance, and securing high crop yields [18], drug release [19], wastewater treatment [20], controlled release of a third-generation EGFR inhibitor [21], and long-term release of favipiravir [22].

Iron compounds can act as catalyst for photosynthesis process; furthermore they are involved in enzyme activity and RNA synthesis [23]. Consequently Fe_2_O_3_ NPs can facilitate intracellular chemical changes and can act as catalysts [24].

Stable colloidal Fe_2_O_3_ NPs were developed via hydrothermal processing, and the developed Fe_2_O_3_ NPs demonstrated stable colloidal particles. Ferric oxide colloid was employed as nano-fertilizer for tomato plant. The main goal of this study was the improvement of tomato plant resistance against *Fusarium* wilt by Fe_2_O_3_ NPs and the assessment of the ISR indicators of treated tomato plants. Fe_2_O_3_ NPs treatments in either (non-infected or infected) plants demonstrated improvements in photosynthetic pigments, osmolytes, and antioxidant enzymes activity. The beneficial effects of Fe_2_O_3_ NPs were extended to increase not only photosynthetic pigments, osmolytes contents but also the activities of peroxidase (POD), polyphenol oxidase (PPO), catalase (CAT) and superoxide dismutase (SOD) enzymes of the healthy and infected tomato plants in comparison with control**.**

## Materials and Methods

### Materials and Instrumentation

Colloidal ferric oxide particles were fabricated via green synthesis technology (Hydrothermal synthesis); this was accomplished via direct conversion of ferric nitrate to ferric oxide. Further details about hydrothermal synthesis of colloidal ferric oxide particles was reported in the following references [25, 26]. The adopted fluids for hydrothermal synthesis are sub-critical or super-critical fluids (ScF) as shown in Supplementary Fig. S1 [27]. Supercritical water (ScW) requires extreme conditions (Tc 374.2 °C, Pc 220.5 bar); Supplementary Fig. S2 demonstrates the phase diagram of water.

At standard conditions K_w_ has the value of 1 × 10^−14^ mol/l [28, 29]. As water approaches its critical point, its dissociation constant increases to about three orders of magnitude; therefore it becomes a suitable solvent for ionic compounds and free radical processing. However, K_w_ decreases dramatically over the critical point [30]. Supplementary Fig. S3 demonstrates the changes in dielectric constant, density, and ionic product of water with temperature at 24 MPa.

The enhanced OH^−^ level at the critical point can be exploited for NPs synthesis. This can be achieved through hydrolysis of metal salt (Eq. 1) immediately followed by a dehydration step (Eq. 2) [29].

1 $${\text{Hydrolysis}}:{\text{ML}}_{{\text{x}}} + {\text{xOH}}^{ - } \to \quad {\text{M}}\left( {{\text{OH}}} \right)_{{\text{x}}} + {\text{xL}}^{ - }$$

2 $${\text{Dehydration}}:~\,{\text{M }}\left( {{\text{OH}}} \right)_{{\text{x}}} \to {\text{MO}}\frac{{\text{x}}}{2} + \frac{{\text{x}}}{2}{\text{H}}_{2} {\text{O}}$$

A schematic for continuous hydrothermal synthesis is demonstrated in Supplementary Fig. S4. In this technique the super-critical water (ScW) flow was instantly mixed with cold metal salt. Nanoparticles are formed at the interface of the two fluids inside the reactor (R).

Ferric oxide NPs were developed via instant mixing of superheated water stream at 350 °C, and 240 bar (Flow A, 20 ml/min), with metal salt precursor (0.05 M ferric nitrate solution) at 25 °C, 240 bar (Flow B, 10 ml/min). Ferric oxide NPs were fabricated in a sustainable manner at the interface of the two streams inside the reactor (R) (Supplementary Fig. S4).

It is widely accepted that mono-dispersed particles were formed as nucleation and subsequent particle growth are the same for all particles. Further details about hydrothermal processing of Fe_2_O_3_ NPs can be found in the following references [25, 26, 31–33].

Surface morphology of colloidal ferric oxide nano-fertilizers was investigated with SEM, JEOL JSM-5600 LV, Japan. Quantification of deposited iron was conducted suing EDAX detector (JEOL JSM-5600 LV, Japan). Crystalline structure of colloidal ferric oxide nano-fertilizers was investigated with XRD (Shimadzu XRD-6000, Japan). Dynamic light scattering (DLS-PSS-NICOMP 380-ZLS particles sized system St. Barbara, California, USA) measurements were conducted to determine the average size distribution of the synthesized nano-fertilizers. In addition, high-resolution transmission electron microscope (HR-TEM, JEM2100, Jeol, Japan) was used as a fundamental tool for investigating the shape, appearance and the average particle size of the prepared nano-fertilizers. Drop coating NPs samples produced HRTEM examinations onto carbon-coated TEM grids after drying by incubation at 37.0 ± 2 °C in an incubator.

### In Vitro Assessment of Antifungal Activity

Agar well diffusion method was applied to study the antifungal activity of the synthesized colloidal ferric oxide nano-fertilizers (Fe_2_O_3_ NPs) according to Parveen, et al*.* [34]*,* with a few modifications. Fungal inoculum was extent systematically on the sterilized solidified potato dextrose agar (PDA) medium. At the same time, five discs 5 mm diameter were loaded with 50 µl of different concentrations of Fe_2_O_3_ NPs with triplicates The plates are kept for 2 h at the fridge to permit diffusion. The culture plates were incubated at 25 °C for 7 days, and the zones of inhibition (ZOI) were observed and measured.

### In Vivo Assessment Efficacy of Fe_2_O_3_ NPs on Tomato Plant

#### Source of *F*. *oxysporum* f. sp. Lycopersici

*F*. *oxysporum* f. sp. Lycopersici RCMB008001 was obtained from Regional Center for mycology et al. Al-Azhar University, then was confirmed by pathogenicity test according to Hibar et al. [35]. The inoculum of the pathogenic fungus *F*. *oxysporum* was prepared according to Buttner et al. [36].

#### Experimental Design

Four-week-old tomato seedlings (*Solanum lycopersicum* 023) were obtained from Agricultural Research Center (ARC), Giza, Egypt. Uniform seedlings were transplanted into plastic pots (30 in diameter) containing a mixture of sand and clay (1: 3 W/W), total 5 kg, in a plastic pot. After the transplant, the seedlings left for 5 days before any treatments with normal irrigation. Afterwards, the inoculum of *F*. *oxysporum* (pathogen) (10^6^) was applied. Two concentrations (10 µg/mL and 20 µg/mL) from colloidal ferric oxide nano-fertilizers (Fe_2_O_3_ NPs) were applied for 3 times (1 time each week; in the period before and after flowering). This experiment was carried out in the garden of Plant and Microbiology Department, Faculty of Science, Al-Azhar University, Cairo, Egypt. The pots were arranged in a completely randomized design with five replicates as follows treatments; 1: control healthy, 2: control infected, 3: healthy treated with Fe_2_O_3_ NPs (10 µg/mL), 4: Infected treated with Fe_2_O_3_ NPs (10 µg/mL), 5: infected treated with Fe_2_O_3_ NPs (20 µg/mL), and 6: infected treated with Fe_2_O_3_ NPs (20 µg/mL). Sixty days old plants have been carefully uprooted and analyzed for the different parameters described below.

#### Disease Symptoms and Disease Index

Disease symptoms were assessed 60 days old and the disease index and protection percent were evaluated according to Farrag et al. [37]. Percent Disease Index (PDI) was calculated using the five-grade scale according to the following formula:

$${\text{PDI}} = \left( {{\text{1n}}_{{1}} + {\text{2n}}_{{2}} + {\text{3n}}_{{3}} + {\text{4n}}_{{4}} } \right){\text{100/4n}}_{{\text{t}}}$$

where n_1_-n_4_ is the number of plants in the indicated classes and nt is the total number of plants tested.

Additionally, Percent Protection (P %) was calculated using the following formula:

$${\text{P}}\% = {{{\text{A}}{-}{\text{B}}} \mathord{\left/ {\vphantom {{{\text{A}}{-}{\text{B}}} {\text{A}}}} \right. \kern-\nulldelimiterspace} {\text{A}}} \times 100\%$$

where A is PDI in infected control plants and B is PDI in infected treated plants.

#### Morphological and Biochemical Resistance Indicators in Tomato Plant

The plant samples were collected for different morphological growth traits (shoot high, root length and number of leaves). Photosynthetic pigments were assayed according to Vernon et al. [38]. The soluble carbohydrate content of the dried shoot was calculated as the method mentioned by Iigoyen et al. [39]. Total protein was determined according to Lowry et al. [40]. Total shoot phenol content was assayed as described by Diaz et al*.* [41]*,* and enzyme activity were determined by the advanced publications [42–44], and [45].

## Results and Discussion

### Characterization of Colloidal Ferric Oxide Nano-Fertilizers

**Fig. 1:**
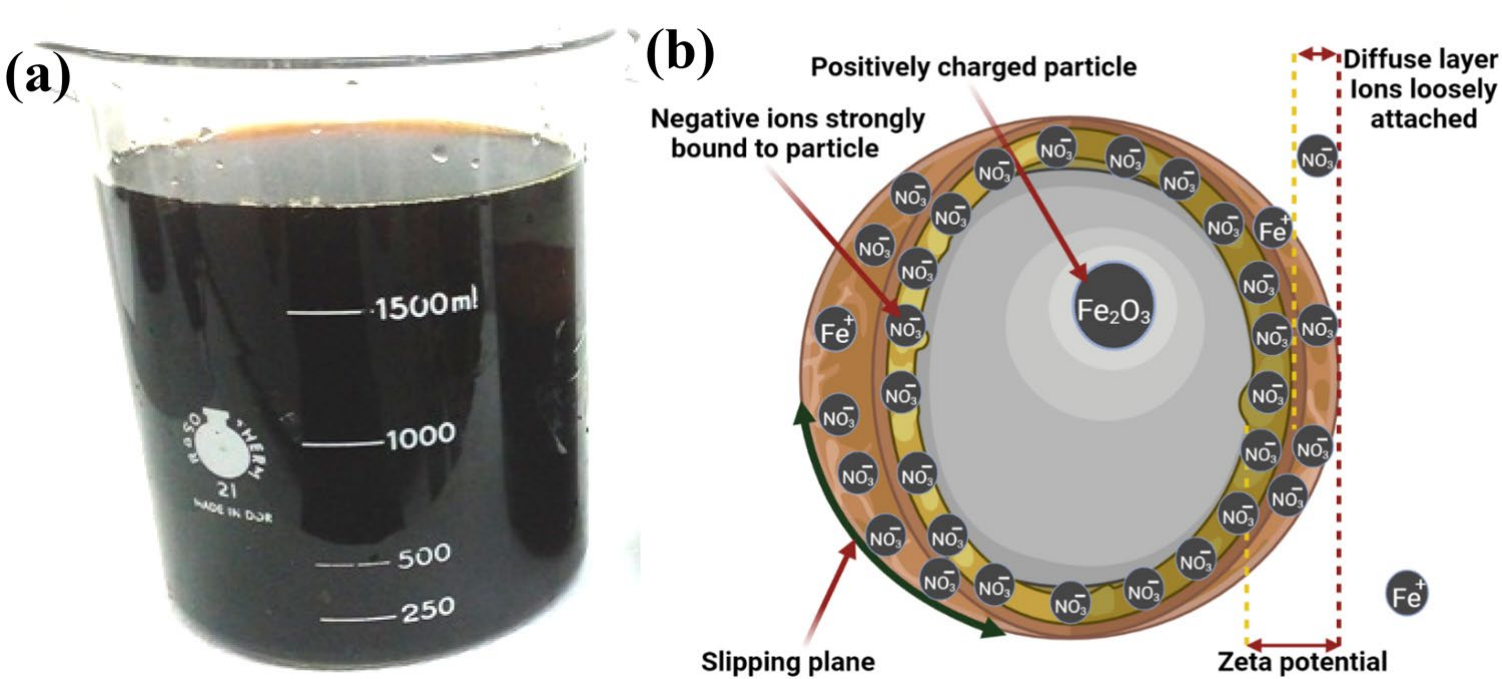
Stabilized Fe_2_O_3_ NPs (**a**), Stabilization mechanism due to electrostatic double layer (**b**)

**Fig. 2:**
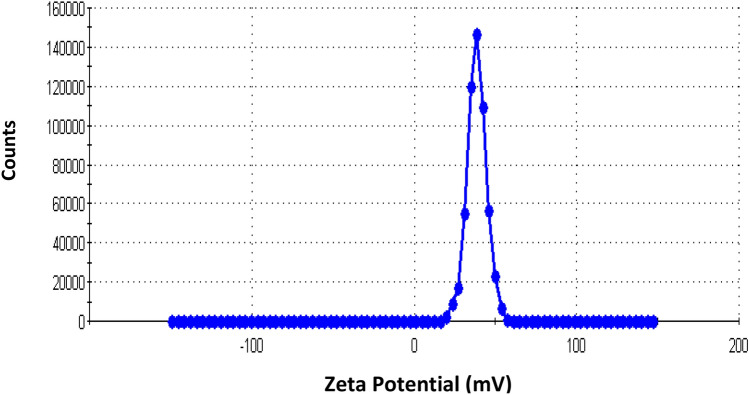
Zeta potential of colloidal ferric oxide

α-Fe_2_O_3_ was manufactured using hydrothermal synthesis technique. The synthesized colloidal particles demonstrated deep red color (Fig. 1a), and the particles did not flocculate with time. Stabilization mechanism was correlated to electrostatic stabilization with nitrate ions (Fig. 1b). The colloidal particles demonstrated Zeta potential value of  +38.5 mV (Fig. 2). Zeta potential confirmed the electrostatic stabilization of colloidal ferric oxide particles.

**Fig. 3:**
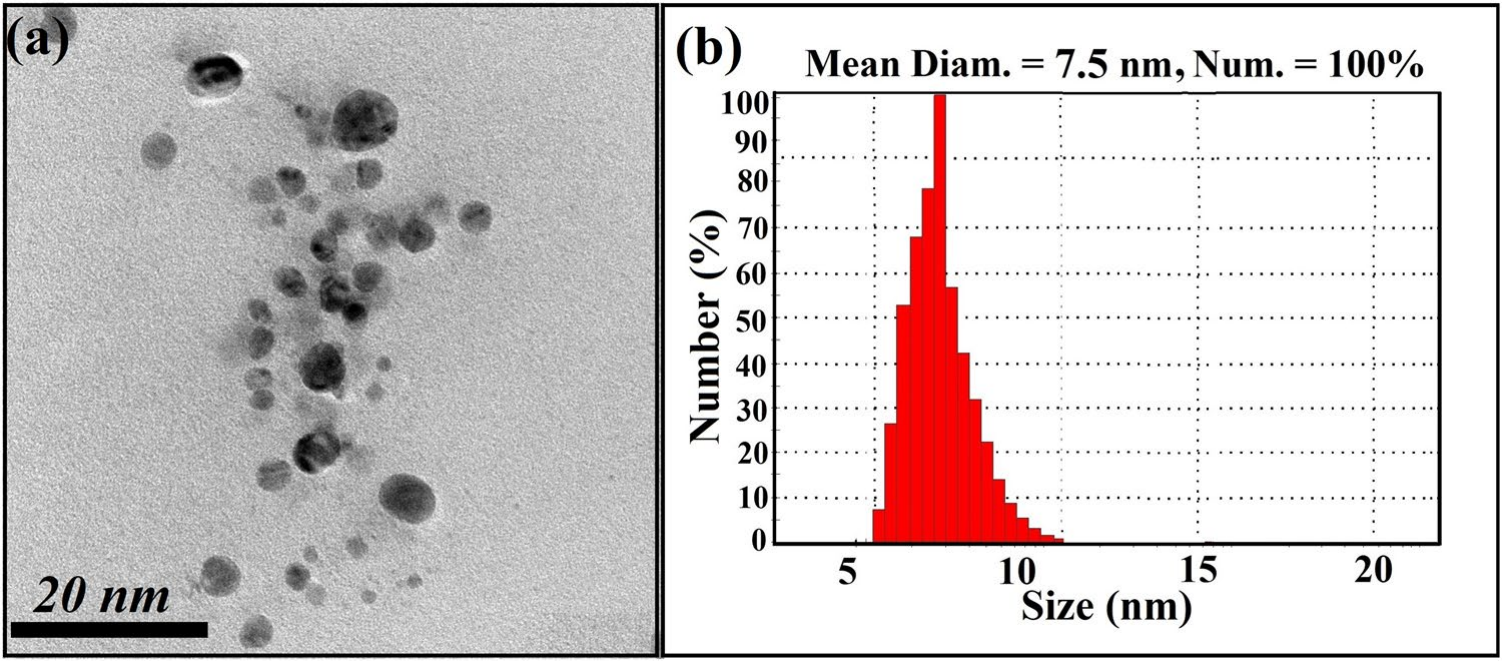
**a** HRTEM micrographs of the synthesized Fe_2_O_3_ NPs, and **b** the average particle size distribution calculated by DLS analysis

HRTEM micrographs demonstrated mono-dispersed Fe_2_O_3_ NPs possesses a spherical shape of 5 nm average particle size (Fig. 3a). HRTEM micrographs confirmed high quality mono-dispersed particles with uniform particle size. On the other hand, particle size distribution was calculated by DLS, and the result indicated that the average Fe_2_O_3_ NPs particle size distribution was found to be 7.5 nm by 100% as displayed in Fig. 3b.

It was noted that, the particle size distribution estimated from DLS analysis was more than the average particle size determination by HRTEM images. The reasons are defined as the DLS method estimated the hydrodynamic radius which founded around the synthesized colloidal Fe_2_O_3_ NPs and enclosed by the water particles regarding the large sizes of the capped Fe_2_O_3_ NPs [46].

**Fig. 4:**
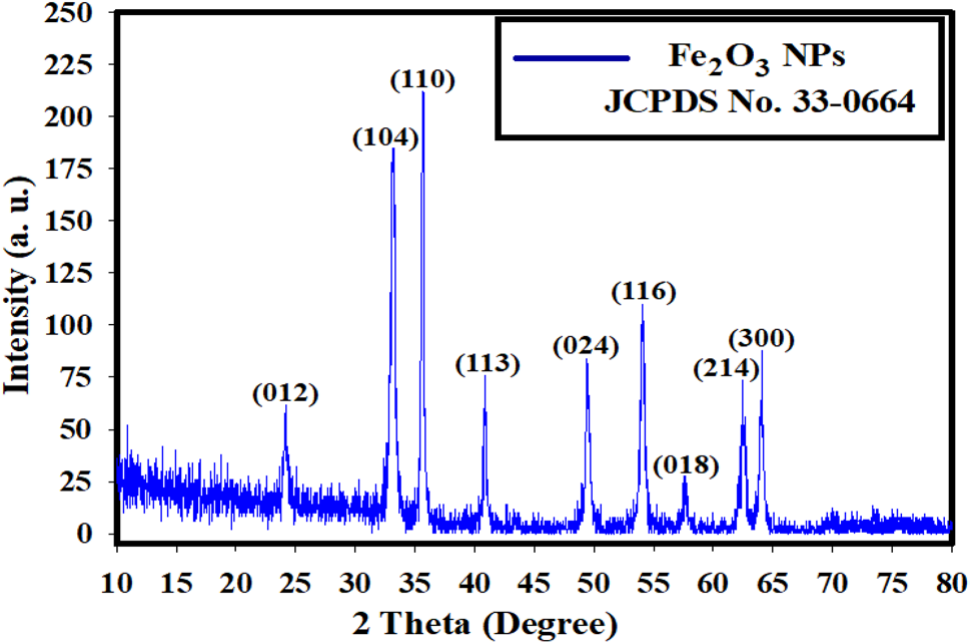
The crystallinity behavior of α-Fe_2_O_3_ NPs by XRD analysis in comparison with the reference code of 33–0664

XRD diffractogram showed high-degree hematite (α-Fe_2_O_3_). XRD system was conducted to study the crystal composition and state of the incorporated Fe_2_O_3_ NPs (Fig. 4). The conducted XRD models agree to the specific α-Fe_2_O_3_ original (JCPDS No. 33-0664). The unique peaks looked at the next 2θ arranges ≈ 24.12°, 33.58°, 35.35°, 40.78°, 49.59°, 54.22°, 57.41°, 62.55°, and 65.62° corresponding to (012, 104, 110, 113, 024, 116, 018, 214, and 300) planes, respectively and showing its standard cubic spinel composition [47]. There are no unknown crystalline phases and impurities in the Fe_2_O_3_ NPs.

This matches with the unique composition of the complete α-Fe_2_O_3_ crystal with a rhombohedral centered hexagonal building (R3c space system) [48–52]. The most important diffraction peak near 35.35° implies that (110) facets remain the dominant α-Fe_2_O_3_ crystal construction with 5.95 nm crystal size according to Williamson-Hall (W–H) equation [53].

**Fig. 5:**
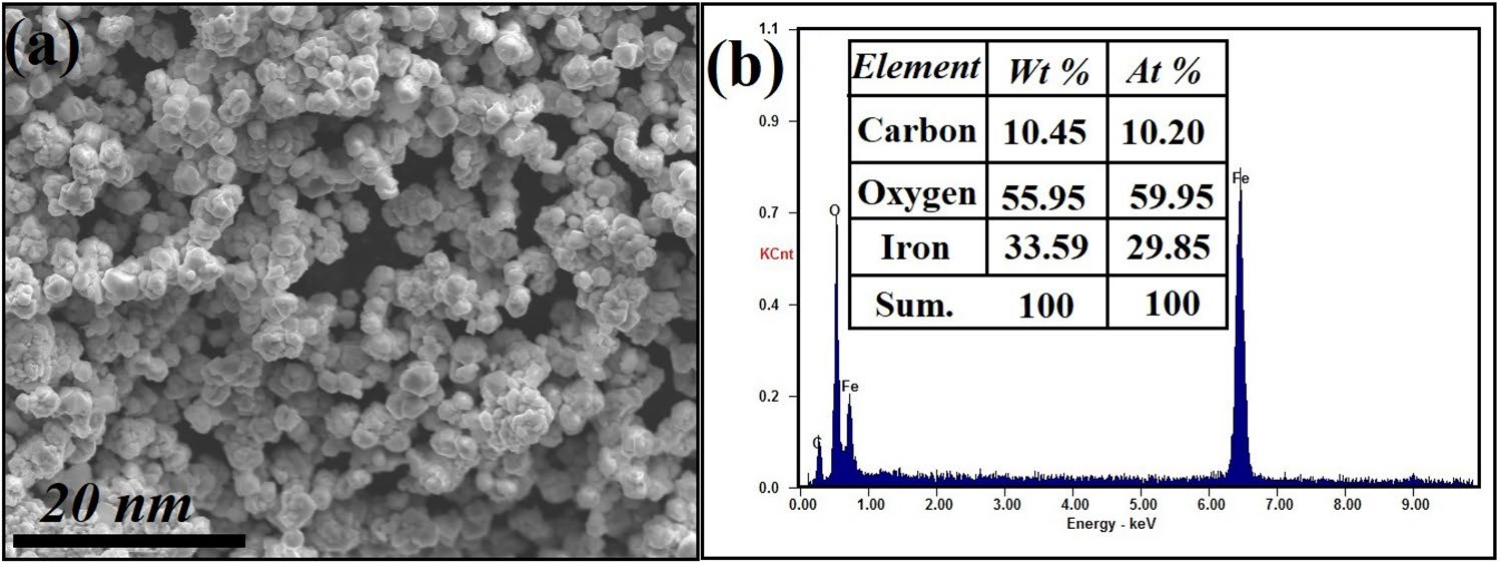
**a** SEM micrographs of the synthesized Fe_2_O_3_ NPs, and **b** EDX elemental analysis of Fe_2_O_3_ NPs powder

The surface morphology, purity, and the elemental composition of the prepared α-Fe_2_O_3_ NPs were studied, as shown in Fig. 5. SEM analysis showed that the prepared α-Fe_2_O_3_ NPs had a semi-spherical structure, with a uniform distribution as displayed in Fig. 5a.

EDX analysis revealed the high purity of the prepared α-Fe_2_O_3_ NPs, as indicated by the presence of atoms characteristic to each component of it (Fe and O atoms) and the absence of foreign atoms that may appear as impurity, Also, carbon atom (C) was corresponded to the holder which used for the SEM imaging process as illustrated in Fig. 5b.

SEM micrographs of dry Fe_2_O_3_ NPs confirmed the vast tendency of NPs to diminish their number and surface area, with dramatic decrease in NPs surface area and reactivity [54, 55]. There is great advantages of using NPs in colloidal state.

### In Vitro Antifungal Potential

Iron oxide nanoparticles showed significant anti-mycotic activity against rot fungal pathogens [56]. Therefore, Fe_2_O_3_ NPs were synthesized in this study to control *F*. *oxysporum*. The antifungal activity of Fe_2_O_3_ NPs was assessed using the well diffusion method [57]; as shown in Supplementary Fig. S5. Results illustrated that Fe_2_O_3_ NPs had antifungal activity against *F*. *oxysporum*.

There are a relationship between the crystal structure (XRD) of the synthesized Fe_2_O_3_ NPs and their anti-fungal properties [58, 59]; the massive surface area and massive reactivity (high interfacial surface area, and small crystal size and structure), therefore increase reactivity of the synthesized nanocomposite against fungal cells [60, 61].

The small size and high surface area increasing the possibility for more interaction with the fungal cells (surface charge will interact more effectively with the fungus) through the charge attraction and diffusion across the fungal cell wall and therefore increasing the antifungal potential [62, 63].

### Control of Wilt Disease Caused by ***F***. ***oxysporum*** by Fe_2_O_3_ NPs (In Vivo)

Disease severity was the first guide to govern systemic resistance in treated plants by Fe_2_O_3_ NPs. Application of Fe_2_O_3_ NPs at concentrations (20 µg/mL and 10 µg/mL) were applicable in decreasing disease index (Table 1). Fe_2_O_3_ NPs (at 20 µg/mL) was active more than 10 µg/mL treatment that reduced percent disease indexes by 15.62 and gave highly protection by 82.15% compared to infected plants that similar to recent studies [64, 65].

**Table 1:** Effect of colloidal Fe_2_O_3_ NPs on the disease index of tomato plant infected with* Fusarium* wilt under pot conditions

Treatment	Disease symptoms classes					DI (disease index) (%)	Protection (%)
	0	1	2	3	4		
Control healthy	8	0	0	0	0	0	–
Control infected	0	0	1	2	5	87.5	0
Infected and treated with Fe_2_O_3_ NPs (10 µg/mL)	3	2	3	0	0	25	71.42
Infected and treated with Fe_2_O_3_ NPs (20 µg/mL)	4	3	1	0	0	15.62	82.15

Our results similar to Ashraf et al. [66], which reported that Fe_3_O_4_ NPs significantly reduced the disease severity in tomato plants infected with *F*. *oxysporum* by an average of 47.8% resulting in increased plant growth variables at exposure to 10 µg/mL of iron oxide NPs. Plants stimulate a toxic oxidative-burst by accumulative iron concentrations to reduce pathogen virulence; roots mutualistic interactions also encounter phytodiseases via iron uptake as well as antagonism for iron achievement produces a systemic resistance that signal mechanisms in roots for iron-uptake [66].

### Morphological Indicators

Morphological features (shoot length, root length, and number of leaves) were significantly decreased due to *Fusariu*m wilt infection. The reduction of all growth parameters showed dangerous losses in plant. In this respect, the drop in growth may be associated with different reasons; among them *Fusariu*m enters through the roots of the plant and proliferates in the vascular tissues leading to breakdown of the water economy of the infected plants [67].

The results indicated that foliar application with Fe_2_O_3_ NPs colloidal solution was great enhancement of growth parameters similar to the literature that discussed NPs to enhance the growth of different crop plants [68].

**Fig. 6:**
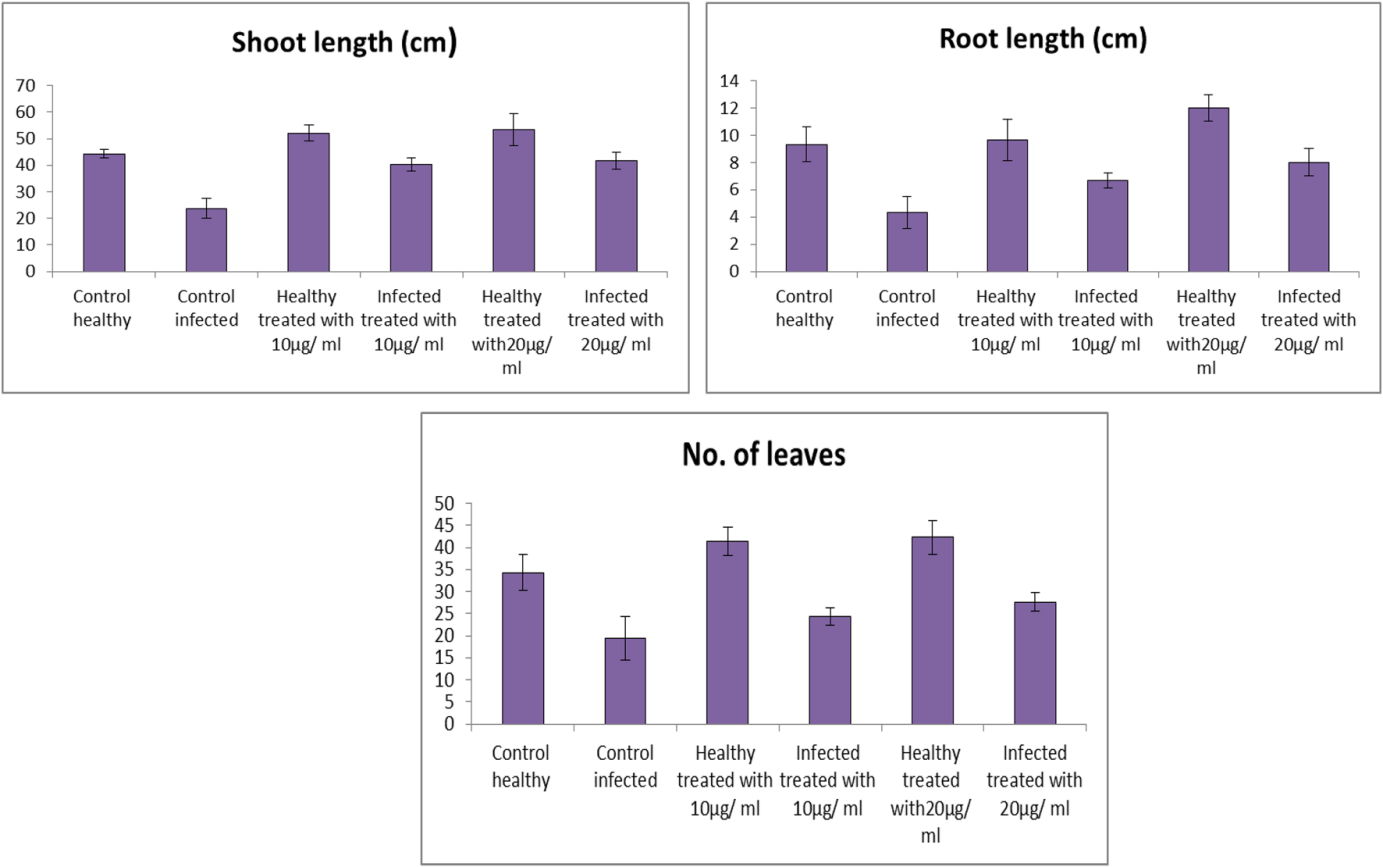
Effect of Fe_2_O_3_ NPs nano-fertilizer on morphological indicators; shoot length, root length, and number of leaves

Concerning the effect of foliar application; Fe_2_O_3_ NPs colloidal solution as foliar spray method on the tested plants (Fig. 6), it was noticed that application with (Fe_2_O_3_ NPs 20 µg/mL, and 10 µg/mL) improved shoot length (by 76.07%, and 70.45%), root length (84.75, and 53.81%) and number of leaves (43.09, and 25.86%), respectively versus infected plants.

Healthy tomato plants treated with Fe_2_O_3_ NPs solutions as foliar spray method (Fe_2_O_3_ NPs 20 µg/mL and 10 µg/mL) showed an improvement in all morphological aspects shoot length by (20.30% and 17.38%), root length (28.61, and 3.53%) and number of leaves (23.30 and 20.39%), respectively when compared to control.

Iron deficiency leads to failure in the production of chlorophyll and yellowing of areas between the veins of the leaves (commonly referred to as iron chlorosis), which leads to dwarfism and a sharp decrease in vegetative growth characteristics. In severe deficiency, leaves become almost pale white due to loss of chlorophyll. Complete leaf fall can occur and buds can die [69].

Interestingly, iron is a micronutrient that plays a vital role in chlorophyll synthesis, carbohydrate production, cell respiration, the chemical reduction of nitrate and sulfate, and in nitrogen assimilation. Stimulating systemic resistance against nutritional deficiency symptoms and pathogens [70].

The current study showed that the concentration of 20 ppm (20 µg/mL) was better than the concentration of 10 ppm (10 µg/mL), as it led to an improvement in vegetative growth characteristics, which means an improvement in chlorophyll and cell respiration.

Results of the present study are similar to the results of other studies in which Fe_3_O_4_ NPs-treated rocket seedlings showed increased shoot elongation after seed germination and have a positive impact on rocket seed germination. Moreover, it is proved that NPs aggregate to root pores and reduce the root hydraulic conductivity by inhibiting the water uptake [71]. It is possible that, the absence of the necessary amount of water causing the elongation in the cells of root [72].

### Photosynthetic Pigments

**Fig. 7:**
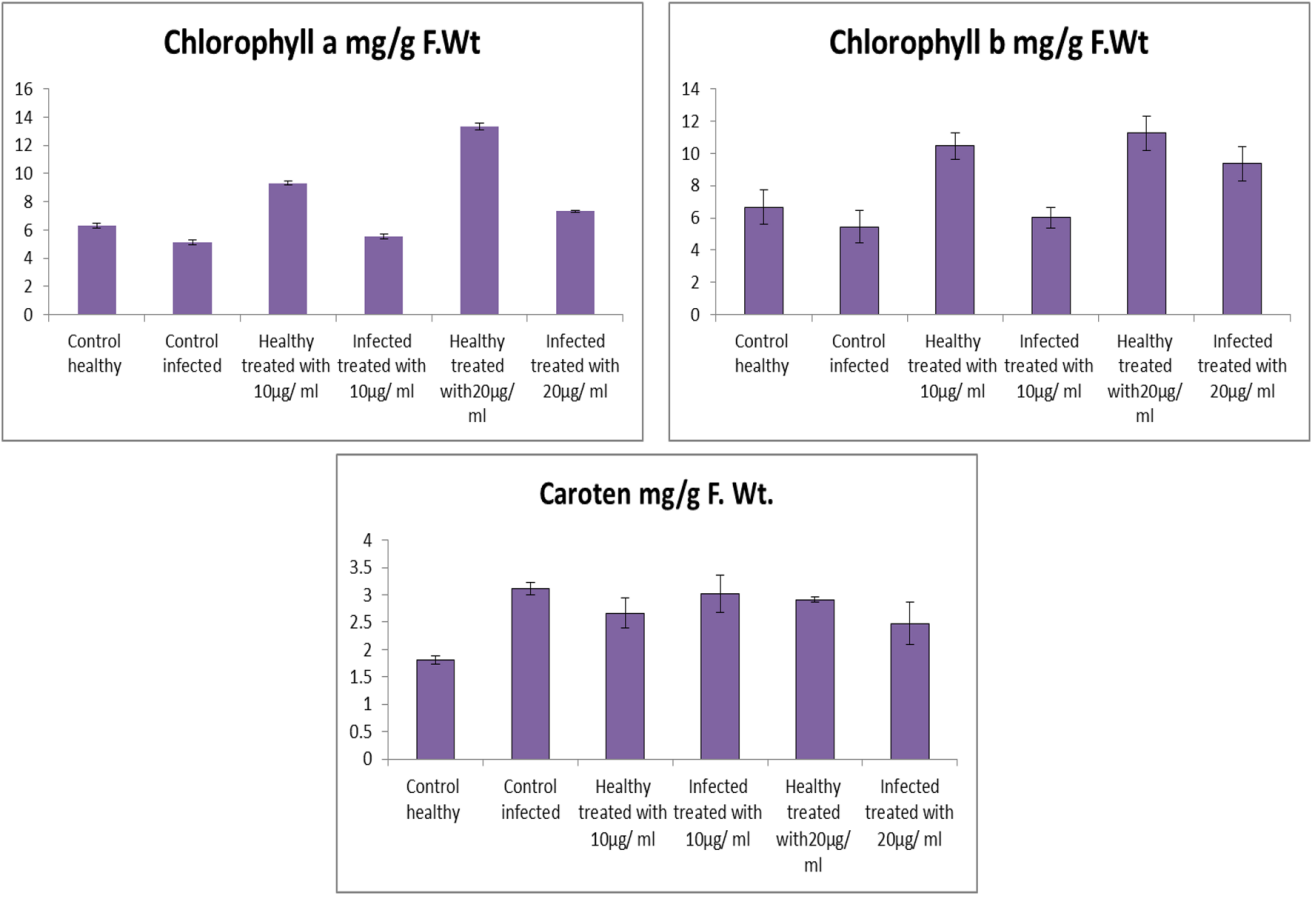
Effect of Fe_2_O_3_ NPs nano-fertilizers on photosynthetic pigments, Chlorophyll a, Chlorophyll b, and Carotenoids

The contents of chlorophyll a and b were significantly decreased in infected plants by 18.95% and 18.44%, respectively as shown in Fig. 7. The decline in chlorophyll may be due to the generation of reactive oxygen species (ROS) causing damage to chlorophyll, which means plants were failed to capture the light and so photosynthesis will decrease or stopped [73].

Additionally, this decrease may be due to chlorophyll deprivation, decreased chlorophyll production and permanency of thylakoid membrane [74]. The present results indicate that, the effects of two concentrations of Fe_2_O_3_ NPs (20, and 10 µg/mL) as foliar spray method, on photosynthetic pigments of tomato plants were investigated. In contrast, there were positive effects of all treatments on plant metabolism. These positive effects may be due to the iron which is a vital nutrient for plants, and its function to take and provide electrons and plays essential functions in the electron-transport chains of photosynthesis and respiration [75, 76].

Foliar fertilization could maintain good plant nutritional status, and iron in particular can be applied to foliage in different chemical forms, including chelates and inorganic iron salts [77].

Results in Fig. 7, indicated that, the contents of carotenoids were significantly increased in tomato plants in response to *Fusarium* infection. Moreover, the obtained results illustrated that the infected plants treated with Fe_2_O_3_ NPs showed a significant decreasing in carotenoid content compared with control infected. It was noticed that application of Fe_2_O_3_ NPs on healthy and infected plants, showed increase in the carotenoid contents. This increase might be attributed to enhanced stomatal conductance, transpiration rate and/or cell size and number [78].

It has been recognized that penetration by foliar-applied iron compounds can occur via cuticle cracks and imperfections and through stomata, leaf hairs, and other specialized epidermal cells. The importance of stomatal versus circular leaf absorption, particularly with regard to aqueous solutions, is a subject of much concern [79].

**Fig. 8:**
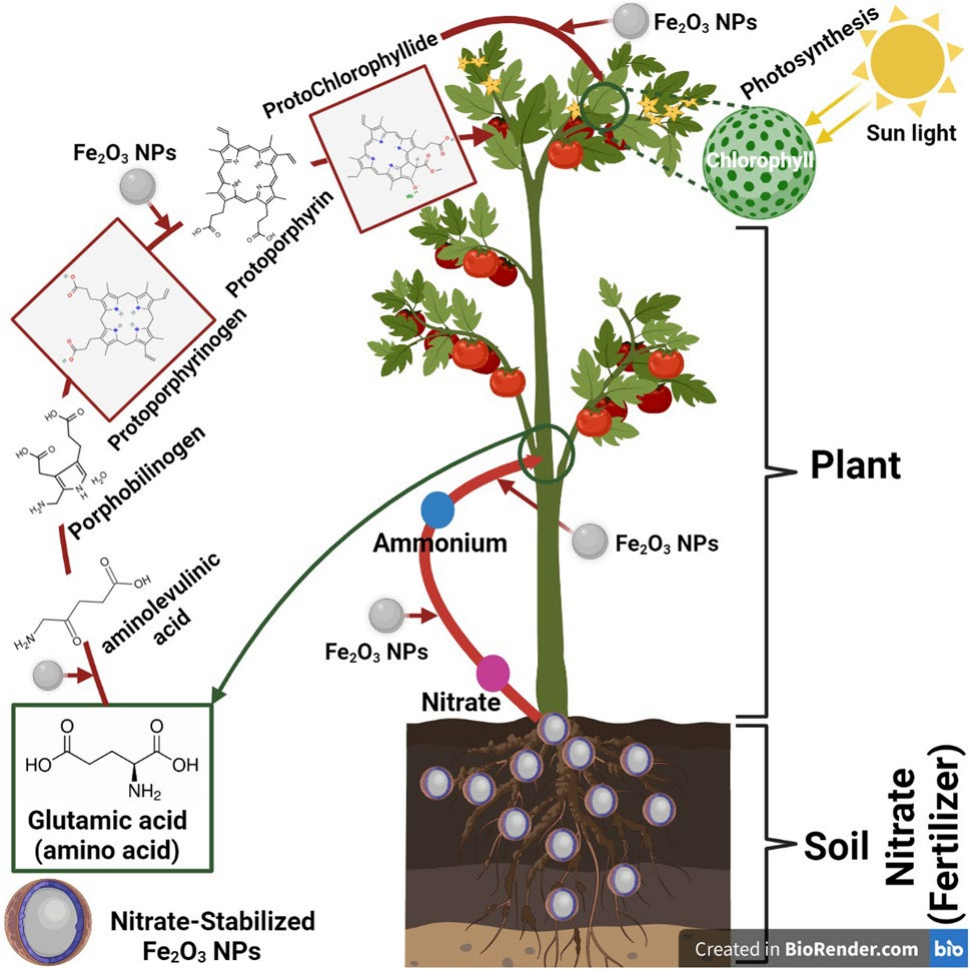
The role of nitrate-stabilized colloidal Fe_2_O_3_ NPs in the creation of chlorophyll and the photosynthetic process in tomato plants

Iron is a vital nutrient for plants, and take and provide electrons and plays essential functions in the electron-transport chains of photosynthesis and respiration [80, 81]. A main portion of iron is confined in chloroplasts where photosynthetic process happens as indicated in Fig. 8. Foliar fertilization could maintain good plant nutritional status, and iron in particular can be applied to foliage in different chemical forms, including chelates and inorganic Fe salts [82].

### Biochemical Resistance Indicators in Tomato Plants

**Fig. 9:**
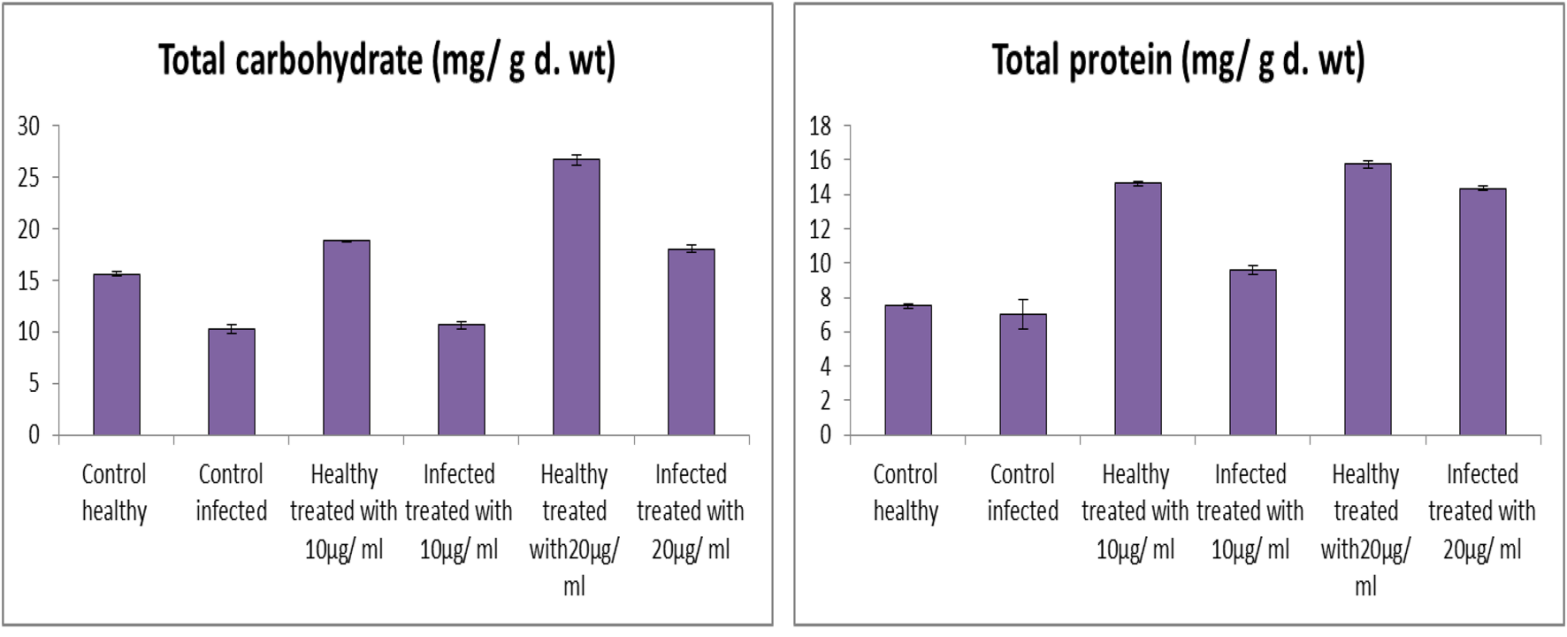
Effect of Fe_2_O_3_ NPs nano-fertilizers on biochemical resistance indicators; total carbohydrate, and total protein

*Fusarium* infected tomato plants showed decreases in contents of soluble carbohydrate, soluble protein by 34.46%, 6.27%, respectively (Fig. 9). In this work, there is a positive correlation between the reduction in osmolytes contents (soluble carbohydrate and soluble protein) and a reduction in photosynthetic pigments and growth of tomato plants in response to *Fusarium* infection. Soluble sugars are involved in the responses to a number of stresses, and act as nutrient and metabolite signaling molecules that activate specific or hormonal-crosstalk transduction pathways, resulting in important modifications of gene expression [83].

On the other hand, foliar application with Fe_2_O_3_ NPs enhanced contents of soluble carbohydrate and soluble protein in shoots of *Fusarium* infected tomato plants when compared to infected plants. The synthesized Fe_2_O_3_ NPs (20 µg/mL) recorded increase in contents of soluble carbohydrate and soluble protein by (43.32%, and 104.27%), respectively compared to infected plants. Healthy tomato plants treated with Fe_2_O_3_ NPs (20 µg/mL, and 10 µg/mL) showed an improvement in soluble carbohydrate by (20.37%, and 70.98%), and soluble protein (95.32%, and 110.41%), respectively when compared to control plants.

In the present work, application of Fe_2_O_3_ NPs solutions as foliar spray method showed increase in the total soluble protein contents in comparison with untreated plants. The continuous accumulations of newly-induced proteins may help in the localization of pathogen infection; the reverse is not true, since the presence of a non-significant amount of induced proteins is a necessary condition to the observed systemic infection. These induced proteins have been defined as pathogenesis related proteins, they implicated in plant defense because of their anti-pathogenic activities [84].

### Phenols Contents in Tomato Plant Leaves

**Fig. 10:**
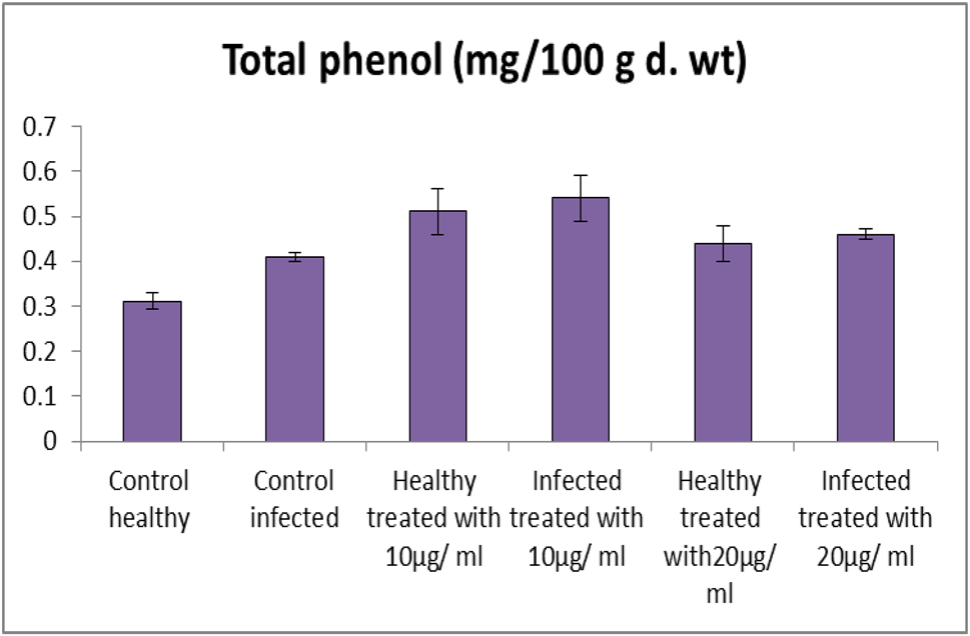
Effect of Fe_2_O_3_ NPs nano-fertilizers on phenols contents in tomato plant leaves

*Fusarium* infected tomato plants exhibited significant increases in the contents of total phenols by 32.25% when compared to control (Fig. 10). In this study, fungal infection increased the contents of total phenols in shoots of tomato plants in accordance with other investigators [73, 85, 86].

Phenolic compounds and ascorbic acid support antioxidant roles by scavenging the free radicals, reducing their reactivity to the membrane components [87]. Moreover, phenolic compounds are also able to stabilize cell membranes by reducing membrane fluidity, which results in reduced mobility of free radicals across membranes, thus limiting membrane peroxidation [88]. Application of Fe_2_O_3_ NPs colloid solutions with different concentration (Fe_2_O_3_ NPs 20 µg/mL, and 10 µg/mL) resulted in a significant increase in the content of total phenols by 12.19% and 31.70%, respectively when compared with infected plants (Fig. 10). Under normal conditions, Fe_2_O_3_ NPs solutions-treated tomato plants (20 µg/mL, and 10 µg/mL) showed an improvement in total phenol content by (41.93%, and 64.51%), respectively comparing to healthy plants. These results accordance with [89]; they reported that iron nanoparticles improved phenol contents of plants.

### Oxidative Enzymes Activity

To obtain more clear indication on some defense-responsible enzymes, mean activities of peroxidase (POD), polyphenol oxidase (PPO), catalase (CAT), and superoxide dismutase (SOD), of the healthy and infected tomato plants were determined in this study.

**Fig. 11:**
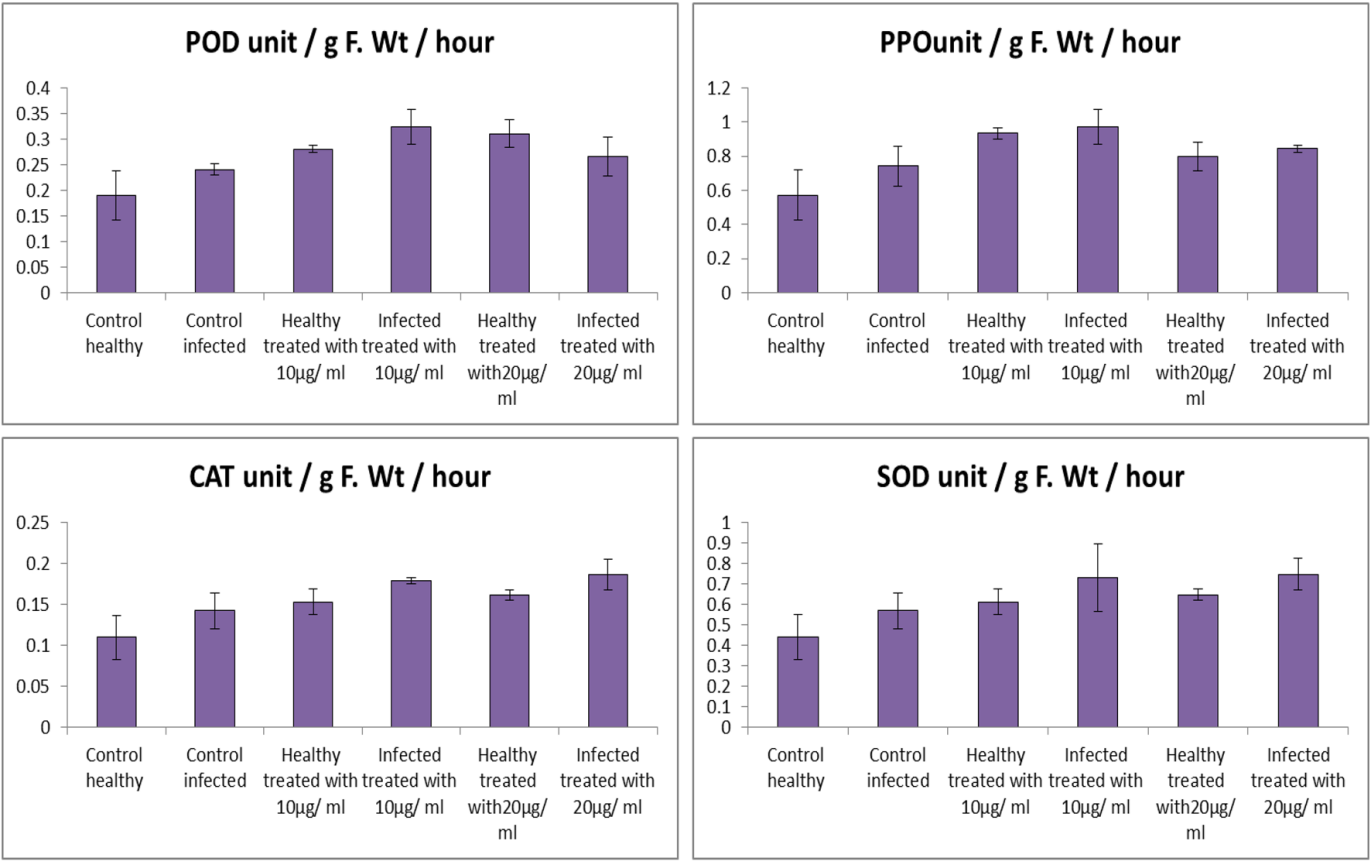
Effect of Fe_2_O_3_ NPs nano-fertilizers on oxidative enzymes activity, POD, PPO, CAT, and SOD

POD, PPO, CAT, and SOD activities were greater in the infected plants as well as plants treated with Fe_2_O_3_ NPs solutions with different concentration (20 µg/mL, and 10 µg/mL). For more, antioxidant enzyme activities provide a large number of defensive enzymes associated with biotic stress [73, 90, 91]. For, peroxidase (POD) and polyphenol oxidase (PPO) activities it was found that, application of Fe_2_O_3_ NPs (10 µg/mL, and 20 µg/mL) on challenged plants increased the activities of POD by (34.43% and 10.37%) and PPO (41.23% and 13.95%), respectively when compared with only infected plants (Fig. 11).

For catalase (CAT), and superoxide dismutase (SOD) activities it was found that, application of Fe_2_O_3_ NPs (20 µg/mL and 10 µg/mL) on challenged plants were increased the activities of CAT by (30.98% and 26.05%) and SOD by (31.33% and 28.69%), respectively compared with infected plants (Fig. 11). Our results showed that antioxidant enzymes activity increased significantly in plants exposed to *Fusarium* infection. The plant showed different strategies to cope with infection as they increase the activity of certain antioxidant enzymes to keep ROS at the lower level in the cell [92]. Nanoparticles can activate anti-stress activities in plants [93].

To eliminate ROS and reduce the toxic effects of stress, plants are equipped with enzymatic antioxidant systems including SOD and POD [94]. According to our results, Fe_2_O_3_ NPs induced higher oxidative stress and higher activity of antioxidant enzymes than untreated plants, which is consistent with literature results [95, 96]. Because iron is involved in enzyme activity and RNA synthesis [97], and due to the high reactivity of the NPs, the synthesized Fe_2_O_3_ NPs can facilitate intracellular chemical changes and can act as catalysts [98, 99]. As in our results, Fe_2_O_3_ NPs in *Cucurbita pepo* had a positive effect on plant growth and increased activity of antioxidant enzymes [100].

**Fig. 12:**
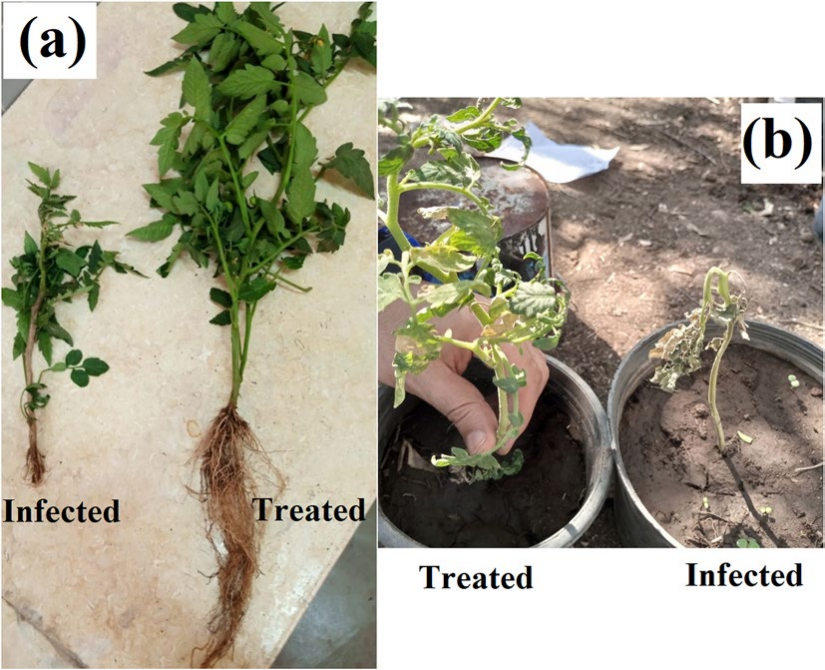
The significant impact of Fe_2_O_3_ NPs on *Fusarium* infected tomato plant, where **a** the comparison between treated and untreated plants regarding the shoot length, root length, and number of leaves and **b** the comparison between treated and untreated plants regarding disease severity and protection %

The significant differences between the healthy treated (Fe_2_O_3_ NPs) tomato plant, and the infected one was investigated (Fig. 12). The effect of *Fusarium* on the plant is represented in Fig. 12a. Symptoms of wilting and a severe decrease in the vegetative total appear; which is reflected on the photosynthesis and all physiological processes. Figure 12b demonstrated an infected plant; that was treated with the fertilizer compound (Fe_2_O_3_ NPs). It is obvious that noticeable improvement in the morphological characteristics, and another infected plant that was not treated showed signs of wilting.

## Conclusion

Stable colloidal ferric oxide particles of 5 nm particle size were efficiently developed by hydrothermal processing. The most important diffraction peak near 35.35° implies that (110) facets remain the dominant α-Fe_2_O_3_ crystal construction with 5.95 nm crystal size according to Williamson-Hall (W-H) equation. SEM analysis showed that the prepared α-Fe_2_O_3_ NPs had a semi-spherical structure, with a uniform distribution. The developed particles were employed as nano-fertilizer for tomato plant against *Fusarium* wilt disease. Application of colloidal Fe_2_O_3_ NPs was applicable in decreasing disease index compared to the infected control. Fe_2_O_3_ NPs (at 20 µg/mL) was the best treatment and reduced percent disease indexes by 15.62 and gave highly protection against disease by 82.15%, and came next Fe_2_O_3_ NPs (10 µg/mL) which reduced percent disease indexes by 25 and have highly protection against disease by 71.42%, related to untreated infected plants. The present results indicate that, the effects of two concentrations of Fe_2_O_3_ NPs (20 µg/mL, and 10 µg/mL) on photosynthetic pigments of tomato plants (healthy & infected) were investigated. We did not observe any photosynthesis inhibition in tomato leaves. The infected plants treated with Fe_2_O_3_ NPs, showed the most potent effect in terms of the length of shoots and roots and the number of leaves per plant. Additionally, tomato plants which treated with Fe_2_O_3_ NPs (20, and 10 µg/mL) showed a significant increase in the content of chlorophyll a and b and carotenoids, total carbohydrates, total soluble proteins, the total phenols ,and antioxidant enzymes activity (POD, PPO, CAT and SOD) compared to the non-treated infected tomato plant. According to the colloidal stability (Zeta results), small size (HRTEM), purity (EDX), promising *in vivo* and *in vitro* results, and high activity in low concentration (20 µg/mL), the bioavailability of the synthesized green nano-fertilizer may be applied in large scale. Additionally, there are some factors must take into consideration for bioavailability of the synthesized green nano-fertilizer such as the stability of nanocomposite in the field conditions, Temp., the acidic or alkaline pH, and the presence of some non-pathogenic microbes in the soil such as PGPR.

## Supplementary Information

Below is the link to the electronic supplementary material.

Supplementary file1 (DOCX 489 KB)

## Data Availability

The datasets supporting the conclusions of this article are included within the article and its additional files.
