# Supplementary material for: Ferric Oxide Colloid: Towards Green Nano-Fertilizer for Tomato Plant with Enhanced Vegetative Growth and Immune Response Against Fusarium Wilt Disease
Source: J Inorg Organomet Polym Mater. 2022 Jul 22;32(11):4270–83. doi: 10.1007/s10904-022-02442-6 (PMC9306234; doi:10.1007/s10904-022-02442-6)
Supplement: Supplementary file 1 — Supplementary file1 (DOCX 489 KB) [file 10904_2022_2442_MOESM1_ESM.docx]

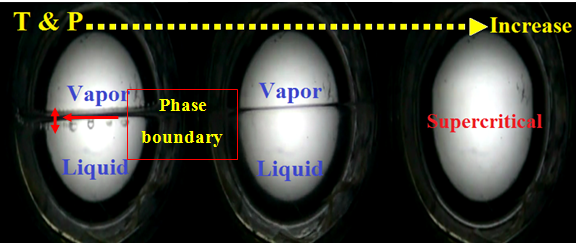


**Fig. S1: Phase change of fluid with pressure and temperature.**


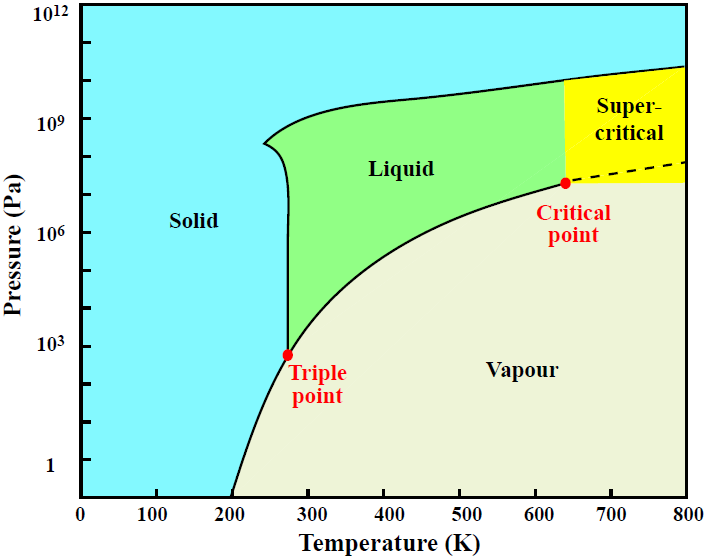


**Fig. S2: Water pressure-temperature phase diagram.**


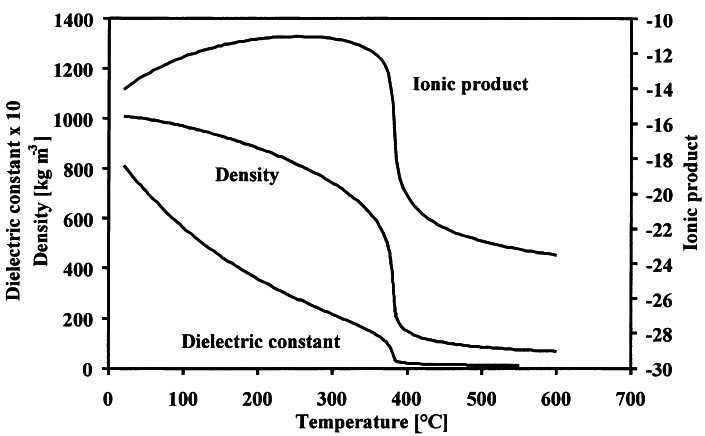


**Fig. S3: Water properties versus temperature at 24 MPa, adapted from ^1^.**


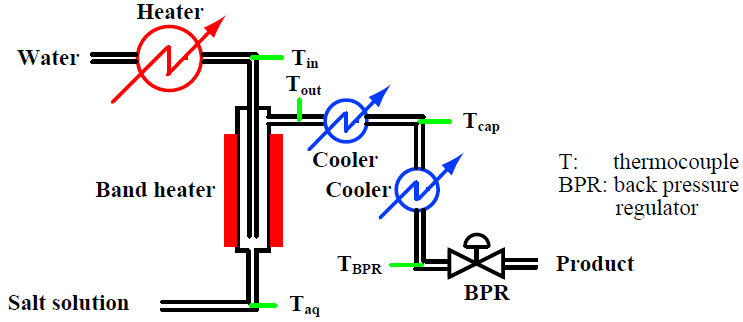

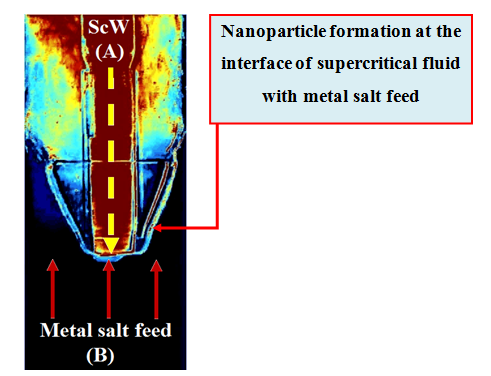


**Flow (A)**

**Flow (B)**

Fig. S4: Flow diagram of the continuous hydrothermal synthesis system used for the instant production Fe_2_O_3_.


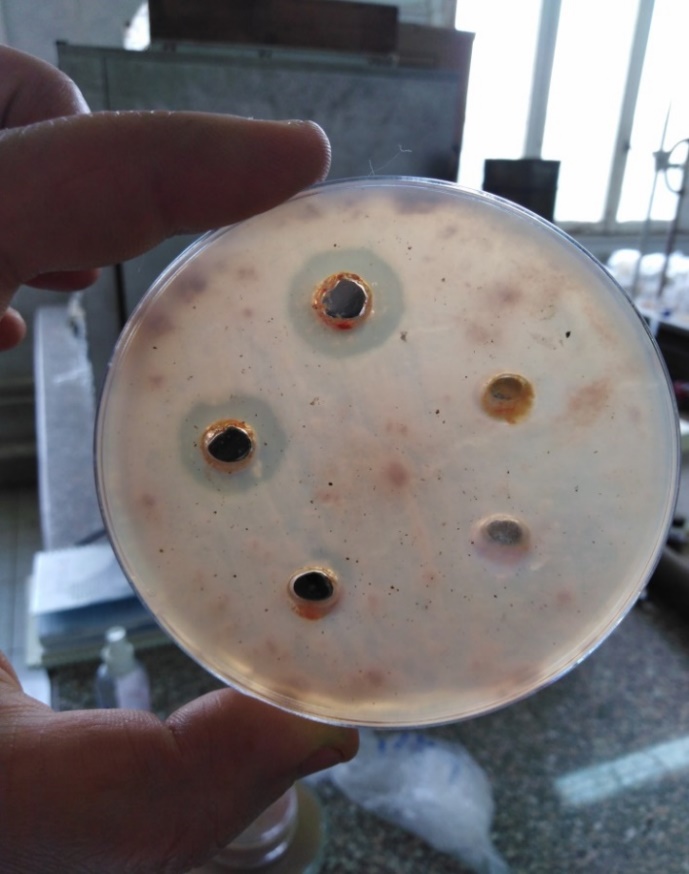


**Fig. S5: Antifungal activity of different concentrations of Fe_2_O_3_ NPs against *F. oxysporum* where clear zones indicating positive results and great activity.**
